# enhancer3D: 3D chromatin structures and enhancer-promoter distance profiles for archaic and modern human genomes

**DOI:** 10.1093/nar/gkaf1256

**Published:** 2025-11-26

**Authors:** Michal Wlasnowolski, Nikita Kozlov, Mikolaj Wojcik, Guy S Jacobs, Dariusz Plewczynski

**Affiliations:** Faculty of Mathematics and Information Science, Warsaw University of Technology, Warsaw 00-662, Poland; Department of Archaeology, University of Cambridge, Cambridge CB2 3DZ, United Kingdom; Faculty of Mathematics and Information Science, Warsaw University of Technology, Warsaw 00-662, Poland; Faculty of Mathematics and Information Science, Warsaw University of Technology, Warsaw 00-662, Poland; Department of Archaeology, University of Cambridge, Cambridge CB2 3DZ, United Kingdom; Faculty of Mathematics and Information Science, Warsaw University of Technology, Warsaw 00-662, Poland; Centre of New Technologies, University of Warsaw, Warsaw 02-097, Poland

## Abstract

Spatial interactions between enhancers and promoters (E–P) are fundamental to gene regulation, yet understanding their evolution, particularly in the context of human divergence and uniqueness, remains a significant challenge. To address this, we present enhancer3D, which is, to our knowledge, the first publicly accessible database of 3D chromatin model ensembles for archaic (Neanderthal and Denisovan) and modern human genomes. The resource enables comparative analysis of E–P spatial distance profiles across evolutionary lineages and between different modern human cell lines (GM12878, HFFc6, and H1-ESC). This facilitates novel investigations into how structural variants and evolutionary divergence impact gene regulation by altering 3D chromatin conformation. For improved user usability, enhancer3D offers an intuitive web interface with integrated visualisation tools, including an embedded IGV genome browser for genomic context and an NGL viewer for interactive exploration of 3D models. All models and datasets are freely available at https://3dgnome.mini.pw.edu.pl/enhancer3D/.

## Graphical Abstract

**Graphical Abstract:**
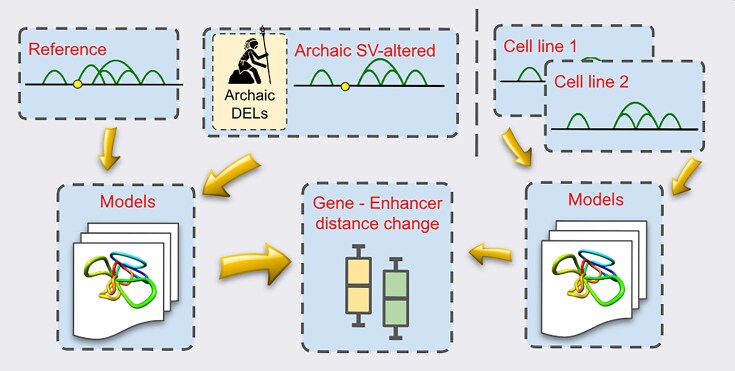

## Introduction

The three-dimensional (3D) organisation of chromatin plays a fundamental role in the regulation of gene expression, primarily by facilitating spatial interactions between enhancers and promoters (E–P) that are often separated by up to 1 Mb along the linear genome [1–4]. These spatial configurations shape cell-type-specific gene expression profiles, and their disruption is increasingly linked to human disease [5–7]. Changes in chromatin architecture caused by structural variants (SVs), such as deletions or duplications, can disrupt regulatory contacts, potentially altering gene expression [8–10]. Such alterations have been implicated in developmental disorders, disease susceptibility [11–14], and evolutionary divergence [15, 16]. For example, it has been proposed that human-specific structural variants (SVs) can reposition Human Accelerated Regions (HARs), altering their 3D proximity to genes and triggering functional rewiring that may have driven their accelerated evolution in humans compared to chimpanzees [17].

Experimental approaches such as Hi-C [18] or ChIA-PET [19] are not feasible for extinct species, making computational modelling a valuable alternative for gaining insight into their 3D genome organisation. Previous studies have applied deep learning to predict Neanderthal chromatin folding directly from DNA sequence [20]. In our work, we propose an alternative approach: by modelling archaic genomes on the basis of high-confidence deletions, we predict differences in 3D structure relative to modern humans. This targeted focus enables fine-resolution modelling of structural changes most likely to affect enhancer–promoter relationships, providing a framework for comparative analysis between archaic and modern human genomes.

**Figure 1.**
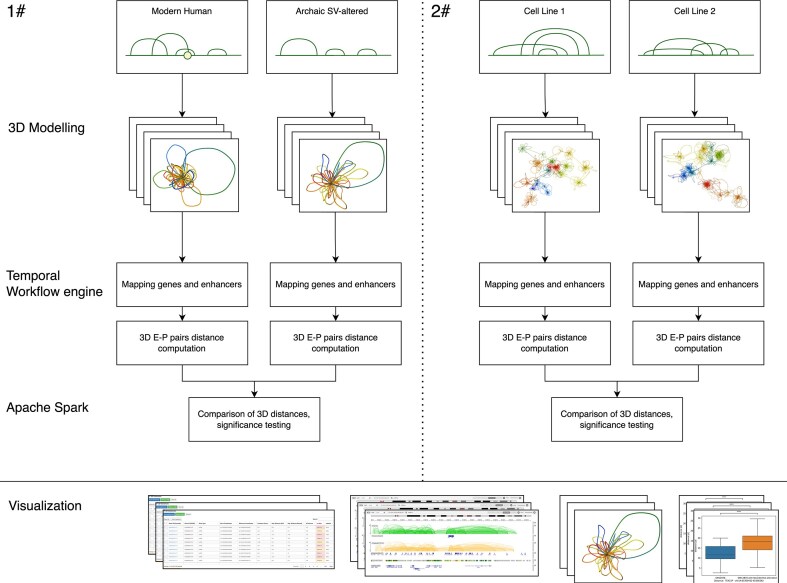
Overview of the enhancer3D data processing pipeline.

In this article, we present enhancer3D (https://3dgnome.mini.pw.edu.pl/enhancer3D/), a public database of tissue-specific 3D chromatin structures. enhancer3D builds upon our previously established 3D-GNOME platform [21], and introduces to our knowledge the first publicly available ensemble of 3D chromatin models for both archaic (Neanderthal and Denisovan) and modern human genomes. enhancer3D represents each region as an ensemble of 3D models, capturing structural variability based on population-averaged contact data. The database is designed to support two key applications: (i) evolutionary analysis of how archaic-specific deletions may have affected chromatin architecture, and (ii) cross-cell-line comparisons of 3D genome organisation in modern humans (Fig. 1). The database consists of two complementary modules. The archaic–modern comparison module focuses on genomic regions (±1 Mbp) surrounding deletions identified in the Simons Genome Diversity Project (SGDP) [22], generating 3D models using CTCF ChIA-PET data from the 4D Nucleome Project (4DN) [23] and the cudaMMC-powered 3D-GNOME 3.0 engine [24]. Models are provided for three biologically distinct cell types—lymphoblastoid (GM12878), embryonic stem cells (H1-ESC), and foreskin fibroblasts (HFFc6)—in both modern human genomes and genomes altered by archaic SVs, enabling comparison of spatial E–P distance profiles across evolutionary lineages and allowing analyses to be performed in a cell-type-specific context. The cross-cell-line module extends this framework to full-chromosome 3D models for the same three cell types in modern humans, integrated with multiple functional genomics datasets, supporting cross-cell-line comparisons of E–P distances.

Enhancer3D is accessible via a user-friendly web interface featuring integrated NGL [25] and IGV [26] viewers. The NGL viewer allows for interactive 3D visualisation of chromatin models, while the IGV browser displays their genomic context, including the locations of SVs, genes, enhancers, and associated data tracks like chromatin states. All structures can be interactively visualised and downloaded in standard CIF format, enabling downstream analysis and reuse. enhancer3D offers a novel and comprehensive platform for studying 3D genome architecture in both regulatory and evolutionary contexts, and is freely available online without registration. We view enhancer3D as a hypothesis-generating resource, enabling systematic identification and prioritisation of candidate loci for targeted validation with orthogonal assays, such as live-cell imaging and genome editing.

## Data sources and processing

### Input data curation

Data curation were performed using the pipeline implemented in our 3D-GNOME 3.0 platform [21]. For our analysis, we utilised *in situ* ChIA-PET data from three cell lines—GM12878, H1-ESC, and HFFC6—provided by the 4DN consortium [23]. We selected only interactions whose anchors overlapped CTCF ChIP–seq peaks, using 3d-analysis-toolkit (https://github.com/SFGLab/3d-analysis-toolkit/).

### Prediction of archaic chromatin interactions

To predict SV-driven changes in chromatin interaction patterns specific to archaic humans, we incorporated biallelic deletions identified in the high-coverage Altai Neanderthal [27] and Altai Denisovan [28] genomes, as distributed by the SGDP, which were converted from hg19 to hg38 coordinates using the UCSC liftOver tool [29]. All analyses were conducted relative to the human reference genome (hg38) with GENCODE v40 annotations [30]. Tissue-specific enhancer locations were obtained from EnhancerAtlas 2.0 [31] and also converted to hg38 coordinates using liftOver.

To predict archaic human chromatin interaction patterns, we used the method of Sadowski *et al.* [8] implemented in the 3D-GNOME platform [21]. This approach rearranges ChIA-PET chromatin interactions observed in modern human cell lines based on specific SVs. The rearrangement process, which considers the impact of the loop-extrusion model, requires the orientation of CTCF anchors. We defined the orientation for each anchor based on the strand containing the CTCF motif with the highest score within its corresponding CTCF ChIP-Seq peak. We performed these predictions separately for the Neanderthal and Denisovan genomes.

### Definition of genomic segments for 3D modelling

#### Full-chromosome models

For full-chromosome modelling in modern humans, we first split each chromosome into segments. Chromatin contact domains (CCDs) were identified using ccd-caller [32], which detects regions with a high density of local interactions. Adjacent CCDs separated by less than 100 kbp were merged using bedtools merge -d 100000. Final segment breakpoints were defined as midpoints between each two neighbour CCDs.

#### Archaic–Modern comparative models

For comparative analyses between archaic and modern genomes, segments were defined around archaic-specific structural variants (SVs). Each segment comprised a 2-Mb window centred on the SV (±1 Mb flanks) to capture potential long-range regulatory interactions. Overlapping segments were merged.

### Generation of 3D model ensembles

#### 3D modelling engine

Chromatin 3D structures were generated using the cudaMMC engine [24], a GPU-accelerated implementation of the 3D-NOME modelling approach originally introduced by Szałaj *et al.* [33] and later incorporated into the 3D-GNOME web server. Specifically, each structure was produced using simulated annealing Monte Carlo polymer modelling, with an energy function that includes terms such as bending, compaction, and distance constraints derived from contact frequency data. The modelling also incorporates orientation constraints on CTCF motifs, consistent with loop extrusion principles, within a hierarchical framework spanning from chromosome-scale through domains down to individual loops. This modelling method has previously been applied in biological studies investigating chromatin folding at specific loci and chromosomal domains [4, 34].

#### Application to the enhancer3D model generation

In the present work, we extend its use to a broader context by generating ensembles of 3D models across three selected cell lines and numerous genomic regions. For each segment, we generated an ensemble of 100 individual 3D models using default parameters.

### Analysis of enhancer–promoter spatial distances

#### Distance calculation

Enhancer and promoter coordinates were mapped onto each of the 100 structures in the ensemble. We measured the 3D Euclidean distance from each promoter’s transcription start site (TSS) to the midpoint of each enhancer. For each segment, we computed the average distance across all enhancer–promoter (E–P) pairs. These distances represent ensemble-averaged spatial estimates derived from polymer models, expressed in arbitrary model units.

#### Archaic vs. modern comparative analysis

To ensure comparability, we restricted the analysis to E–P pairs for which both elements were located outside the SV region. Distributions of E–P distances from the archaic and modern ensembles (100 models each) were compared using the Mann–Whitney U test, with Bonferroni correction for multiple testing.

#### Cross-tissue comparative analysis

Direct comparison of raw distances across cell lines is complicated by variability in segment characteristics and interaction density. To address this, we developed a non-parametric, quantile-based approach. Within each segment, E–P distances were converted into quantile ranks, allowing for relative comparisons across cell types. We focused on enhancers exhibiting large shifts in quantile ranks, specifically those transitioning between the short-range (<33rd percentile) and long-range (>66th percentile) distance categories.

## Database architecture

enhancer3D follows a lakehouse-style design that cleanly decouples storage from compute. All analysis-ready enhancer-promoter (E-P) metrics are materialised as a partitioned, structured dataset stored as Parquet on an S3-compatible object store, MinIO (https://min.io/). Operationally, Temporal (https://temporal.io/) handles fan-out/fan-in workflows (per model ensemble tasks, Enhancer Atlas integration, distance computation), while Apache Spark (https://spark.apache.org/) remains the workhorse for analytic queries and cross-dataset comparisons.

Partitions are organised to mirror dominant access patterns: by ensemble/cell line and genomic window (chromosome and segment boundaries) and, where useful, by analysis mode (regional versus whole-chromosome). This layout aligns with how operators interact with the system (gene name or genomic interval), enabling fast predicate pushdown and partition pruning for gene or region-centric lookups. The result is a modular, schema-evolving data plane: adding a new cell line, archaic assembly, or annotation is a matter of dropping new Parquet partitions or side tables into MinIO, after which the same distributed “join-and-filter” path produces updated visualisations without replatforming.

This separation keeps ingestion resilient to heterogeneous inputs and lets the compute tier scale elastically with query load. Because both core data (E-P Parquet) and contextual datasets live in the object store, the architecture stays cloud-portable and future-proof.

## Web interface

The homepage of enhancer3D features a selection form that allows users to choose between two main types of comparative analyses:

### Archaic versus modern human analysis

This mode allows users to compare 3D chromatin interactions in regions affected by structural variants (SVs) specific to archaic humans (Neanderthals or Denisovans) against the modern human reference. The analysis begins by selecting one of the available modern human cell lines (GM12878, H1-ESC, or HFFc6). After submitting the query, the system identifies all precomputed modelling segments overlapping the queried region. Users are then presented with a list of matching segments. Selecting one of them opens the corresponding results and visualisation page.

### Cross-cell line analysis

This mode enables the comparison of chromatin interaction patterns between two different modern human cell lines, selected from the three lines mentioned above.

Once the analysis type is selected, users specify a genomic region of interest by entering coordinates (chr:start-end) or providing a gene name. If a gene name is provided, the system automatically resolves it to the corresponding genomic coordinates. A Load Example button is also provided to populate the form with a sample query. For cross-cell-line comparisons, the visualisation can be performed at the full-chromosomal level as well.

## Data visualisation

### Genomic browser visualisation

**Figure 2.**
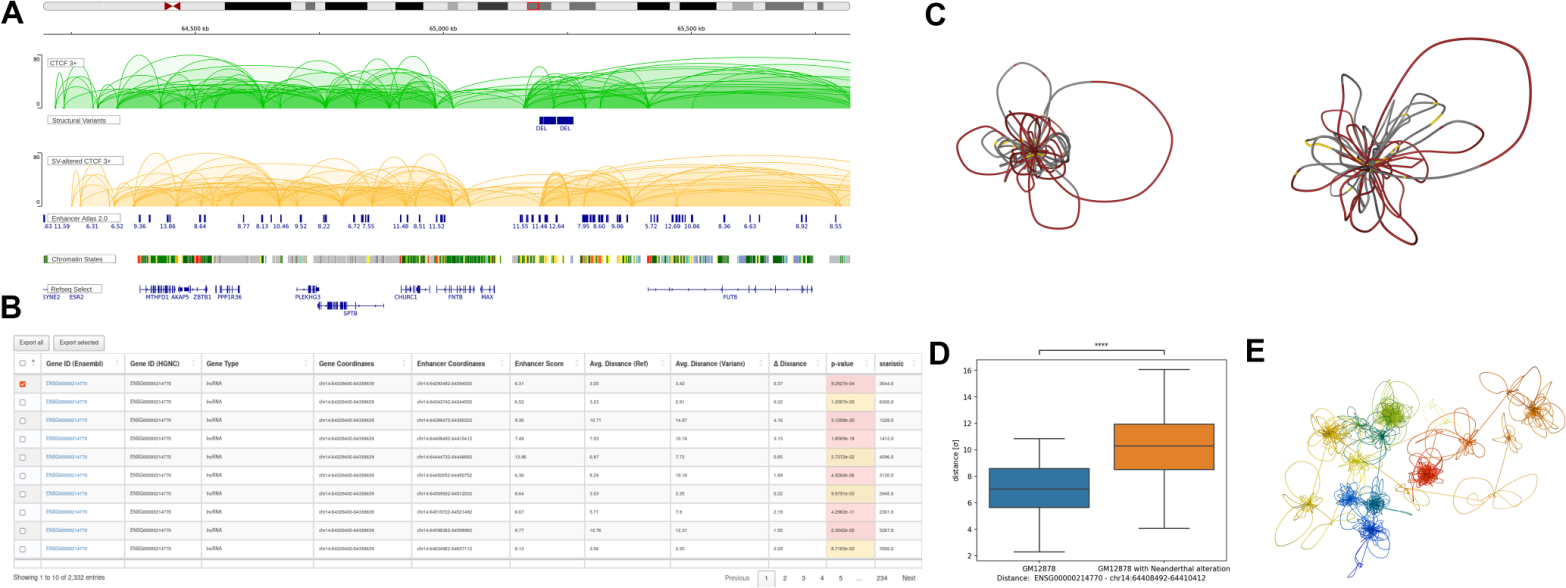
Results for the region chr14:65 139 963–65419356. (**A**) Screenshot from the IGV browser showing CTCF-mediated chromatin interactions in the reference GM12878 cell line (green) and in the GM12878 cell line altered by Neanderthal SVs (gold), together with genomic annotations for the selected region (SVs, enhancers, chromatin states, and genes). (**B**) Responsive table showing the distances between genes and enhancers in the reference GM12878 cell line and in the SV-altered GM12878 cell line. (**C**) 3D chromatin models reconstructed for the selected region for the reference GM12878 cell line (left) and the SV-altered GM12878 cell line (right), based on CTCF interactions. (**D**) Box plot showing the distance distribution between the ENSG00000214770 gene and an enhancer located in the chr14:64 408 492–64 410 412 region for the reference and SV-altered genomes (ns *P* > 0.05 (not significant); *P ≤ 0.05; **P ≤ 0.01; ***P ≤ 0.001; ^****^P ≤ 0.0001). (**E**) 3D chromatin model of chromosome 14 of the GM12878 cell line.

Datasets are visualised using an embedded IGV [26], which displays ChIA-PET interactions, chromatin states for modern cell lines, as well as annotated locations of enhancers, genes, and archaic SVs (Fig. 2A). To support exploration, the IGV panel accepts user-provided BED, BEDPE, or VCF tracks for direct overlay with the displayed datasets.

### Promoter–Enhancer distance comparison

Results of E–P distance analyses are presented in an interactive, JavaScript-based results table. The table includes gene identifiers (linked to Ensembl [35]), genomic coordinates of genes and enhancers, and enhancer scores (Fig. 2B). Users can sort, filter, and search entries by any column, including filtering based on significance thresholds (*P*-value < 0.05). Selected entries can be exported in CSV format.

In the Archaic vs Modern analysis mode, the table displays average E–P distances for both the modern (reference) and archaic (SV-altered) model ensembles, along with the calculated difference. Additionally, users can generate boxplots comparing E–P distance distributions for selected gene–enhancer pairs by selecting entries and clicking the Generate distance boxplots button. The resulting boxplots are displayed below the table and are available for download in high-resolution format. In the Cross-Cell Line mode, changes in E–P distances are quantified as shifts in the quantile of the spatially closest enhancer relative to its gene promoter.

### 3D model visualisation

To explore 3D chromatin models interactively, users can click the Open 3D View button on the results page, which opens an integrated NGL viewer displaying two chromatin models side-by-side: either the modern and archaic structures (Archaic vs Modern mode) or models from two selected modern human cell lines (Cross-Cell Line mode) (Fig. 2C and E). Genes and enhancers are visually distinguished by colour and size. All displayed models can be downloaded from the Download section in CIF format for use with local molecular visualisation tools such as UCSF Chimera [36].

## Conclusions and future directions

enhancer3D makes two primary contributions. First, it provides, to our knowledge, the first publicly available database of predicted 3D chromatin models for archaic hominins, enabling comparative analyses of genome architecture across evolutionary lineages. Second, it offers a framework for cross-cell-line analysis in modern humans, facilitating studies of tissue-specific gene regulation. Together, these modules create a comprehensive resource for exploring how 3D genome architecture shapes both human evolution and cell-type identity. Equipped with interactive visualisation tools such as the embedded NGL 3D viewer and the IGV genome browser, enhancer3D offers a powerful platform for data exploration, making it an accessible tool for the broader scientific community.

In this initial release, enhancer3D incorporates three biologically distinct modern human cell lines, selected on the basis of high-quality CTCF ChIA-PET datasets from the 4DN consortium: lymphoblastoid GM12878, embryonic stem cell H1-ESC, and fibroblast HFFc6. Although this limited panel cannot capture the full breadth of human regulatory variation, it provides a proof-of-principle comparative framework across both evolutionary lineages and cell types.

As a direct experimental study of human–archaic 3D genome differences is not feasible, enhancer3D focuses on high-confidence deletions where predicted effects on chromatin folding are most robust. Future extensions may incorporate additional types of variation, broadening the scope of comparative analyses.

Additionally, future developments will include several key expansions. We intend to incorporate additional high-quality archaic genomes as they become available and expand the collection of modern human cell lines to improve representativeness and support broader comparative analyses. The next extension will incorporate additional regulatory layers, such as epigenomic data, to enable more comprehensive analyses of transcriptional regulation. Planned datasets include histone modification profiles from modern human tissues and reconstructed DNA methylation maps of archaic human genomes [37]. Building on this, we plan to develop dedicated functionalities to analyze the impact of archaic introgression on the 3D genome of modern humans, creating opportunities to investigate how introgressed variants may contribute to disease susceptibility and to identify potential therapeutic targets [38–40]. Such loci will be prioritised for further *in silico* analyses and experimental validation to test their impact on 3D genome structure and gene expression in modern human cellular contexts. In addition, incorporating high-quality CTCF datasets from non-human primates (e.g. chimpanzee, macaque) [16] will enable direct cross-species comparisons of chromatin architecture and gene regulation, further broadening the evolutionary scope of enhancer3D.

We plan to use the enhancer3D E-P distance distributions as a performance benchmark for evaluating chromatin 3D modelling engines [24, 32, 41]. Finally, we aim to extend our modelling pipeline to support high-resolution 3D reconstructions from Hi-C data, broadening the applicability of enhancer3D to a wider range of species and datasets.

## Data Availability

The data underlying this article are available in the enhancer3D database at https://3dgnome.mini.pw.edu.pl/download_enhancer3D/.
